# Differences between the Glycosylation Patterns of Haptoglobin Isolated from Skin Scales and Plasma of Psoriatic Patients

**DOI:** 10.1371/journal.pone.0052040

**Published:** 2012-12-18

**Authors:** Bernardetta Maresca, Luisa Cigliano, Maria Stefania Spagnuolo, Fabrizio Dal Piaz, Maria M. Corsaro, Nicola Balato, Massimiliano Nino, Anna Balato, Fabio Ayala, Paolo Abrescia

**Affiliations:** 1 Dipartimento delle Scienze Biologiche, Università di Napoli Federico II, Napoli, Italia; 2 Istituto per il Sistema Produzione Animale in Ambiente Mediterraneo, Consiglio Nazionale delle Ricerche, Napoli, Italia; 3 Dipartimento di Scienze Farmaceutiche e Biomediche, Università degli Studi di Salerno, Fisciano (Salerno), Italia; 4 Dipartimento di Chimica Organica e Biochimica, Università di Napoli Federico II, Complesso Universitario M. S. Angelo, Napoli, Italia; 5 Dipartimento di Patologia Sistematica - Sezione di Dermatologia, Università di Napoli Federico II, Napoli, Italia; Faculdade de Medicina, Universidade de São Paulo, Brazil

## Abstract

Improved diagnosis of psoriasis, by new biomarkers, is required for evaluating the progression rate of the disease and the response to treatment. Haptoglobin (Hpt), a glycoprotein secreted by hepatocytes and other types of cells including keratinocytes, was found with glycan changes in psoriasis and other diseases. We previously reported that Hpt isolated from plasma of psoriatic patients is more fucosylated than Hpt of healthy subjects. The aim of this study was to compare the glycosylation pattern of Hpt isolated from skin scales or plasma of patients with psoriasis with that of Hpt from cornified epidermal layer or plasma of healthy subjects. High performance liquid chromatography analysis of the glycans isolated from the protein backbone revealed that glycan patterns from skin and plasma of patients were similar, and mostly displayed quantitative rather than qualitative differences from normal pattern. Biotin-labeled lectins were used to evaluate quantitative differences in the glycoforms of Hpt from plasma and psoriatic skin scales. Hpt from skin and plasma of patients showed more fucosylated and branched glycans than Hpt from plasma of healthy subjects. Tryptic glycopeptides of Hpt were also analyzed by mass spectrometry, and a decreased amount of sialylated glycan chains was found in glycopeptides of skin Hpt, as compared with Hpt from plasma. High levels of glycans with fucosylated and tetra-antennary chains were detected on the peptide NLFLNHSENATAK from Hpt of psoriatic patients. Our data demonstrate that specific changes in glycan structures of Hpt, such as enhanced glycan branching and fucose content, are associated with psoriasis, and that differences between circulating and skin Hpt do exist. A lower extent of glycan fucosylation and branching was found in Hpt from plasma of patients in disease remission. Altered glycoforms might reflect changes of Hpt function in the skin, and could be used as markers of the disease.

## Introduction

Haptoglobin (Hpt) is an acute-phase glycoprotein known to bind free haemoglobin (Hb) for degradation and iron recycling [1], [2]. Hpt is produced mostly in liver by hepatocytes [3], [4] and secreted into blood circulation. Its levels markedly increase during the acute phase of inflammation and in neoplastic disease in response to inflammatory cytokines [1]. In addition to binding Hb, a number of other physiological roles of Hpt were suggested. Hpt might play a role in angiogenesis and wound healing, as it inhibits gelatinases thus contributing to remodel the extracellular matrix [5]. Moreover, Hpt was recently reported to bind the apolipoprotein (Apo) A–I and ApoE, and impair their key function in stimulating the enzyme lecithin:cholesterol acyl transferase (LCAT) and mediating cholesterol delivery to hepatocytes [6], [7]. Although the tissue-specific expression of Hpt in some peripheral organs was demonstrated [8]–[12], the role of Hpt in the skin or skin diseases like psoriasis has not yet been studied. Limited studies provide evidence that Hpt might be synthesized and/or secreted into the skin [13], and demonstrate its inhibitory effect on the differentiation of immature epidermal Langerhans cells in antigen presenting cells [14]. Locally produced Hpt might have a modulatory role on skin cells and/or on cells of the immune system, recruited at the site of inflammation. We have previously demonstrated that, in Psoriasis vulgaris, plasma Hpt displays glycoforms with reduced affinity for both Hb and ApoA-I as compared with glycoforms isolated from plasma of healthy subjects, and inhibits the LCAT activity less than normal protein [15]. These glycoforms were suggested to be associated with skin disease and secreted at enhanced levels during inflammation [15], [16]. Actually, abnormal glycosylation of glycoproteins has been correlated with cancer, inflammatory diseases, and congenital disorders [17]. Four asparagine residues of the Hpt subunit β are known to link glycans (N23, N46, N50, and N80) [18], and tri- or tetra-antennary glycans were found on this subunit from patients with rheumatoid arthritis [19], endometriosis [20], or ovarian cancer [21]. In addition, the levels of N-acetylneuraminic acid (NeuAc, also called sialic acid and indicated by the acronym S) and/or fucose (Fuc) were found associated with prostate cancer [22], pancreatic cancer [23], carbohydrate-deficient glycoprotein syndrome [24], or liver disease [25]. We recently reported that the glycan pattern of Hpt isolated from plasma of patients with acute coronary syndrome displays more branched and fucosylated structures as compared to that of Hpt from healthy subjects [26]. We also found higher number of different fucosylated and tri-antennary or tetra-antennary glycans in Hpt from plasma of patients with psoriasis than in controls [16].

Our objective was to study whether the Hpt glycan changes found in the plasma of patients with psoriasis are a consequence of systemic inflammation or can be also found in Hpt isolated from skin lesions. Moreover we compared glycan structures associated with Hpt isolated from skin scales of patients and epidermal layers of healthy subjects, in order to investigate whether specific glycoforms in the skin of patients do exist.

## Results

### HPLC analysis of Hpt glycans

**Figure 1:**
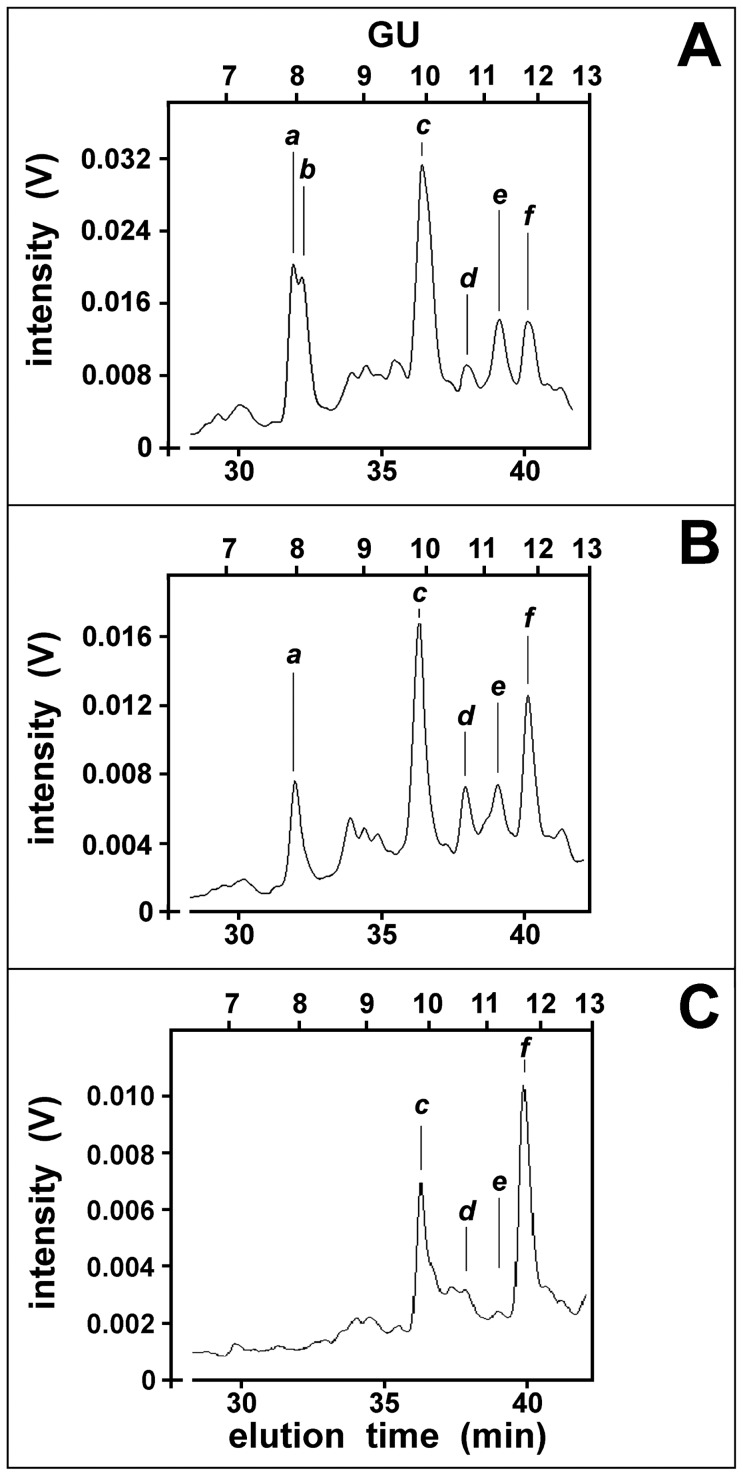
Normal phase HPLC pattern of 2AB-glycans from pHpt-N, pHpt-P and sHpt-P. Purified pHpt-N, pHpt-P, and sHpt-P were deglycosylated by treatment with PNGase F, and their glycans were labelled by 2 aminobenzamide. After solid phase extraction, the glycans were fractionated by HPLC using a TSK-gel Amide-80 column (4.6×250 mm) with a linear gradient of ammonium formate at pH 4.4 (87.5 to 162.5 mM) with CH_3_CN (65 to 35%) in 75 min, at 0.4 ml/min flow rate. Elution was monitored by measuring the label fluorescence at 425 nm (λ_EX_ = 360 nm). Panel A: glycans from pHpt-N. Panel B: glycans from pHpt-P. Panel C: glycans from sHpt-P. The GU ladder represents the migration of labelled oligosacharides with different units of glucose. The marked peaks were used for comparative analysis.

Hpt was purified from plasma (pHpt-P) and skin scales (sHpt-P) of patients with psoriasis, or plasma of healthy donors (pHpt-N). Purified pHpt-P, sHpt-P, and pHpt-N were treated with N-glycosidase to separate N-linked glycans from the polypeptide backbone. The released glycans were labelled with the fluorophore 2-AB and analyzed by HPLC, as previously reported [27]. The elution patterns of the glycans from pHpt-P and sHpt-P were similar to that of glycans from pHpt-N. Six major (namely peaks ***a*** to ***f***) and a number of minor peaks were present on chromatograms of glycans from pHpt-N (Figure 1, panel A). The pattern of pHpt-P glycans was similar to that of pHpt-N glycans, but peak ***b*** was missing (Figure 1, panel B), whereas only peaks ***c*** and ***f*** could be clearly detected in the glycan pattern from sHpt-P (Figure 1, panel C). Differences in maximum and area were found for peaks with the same migration (expressed in GU value). Quantitative measurements of the relative amount of the major peaks were done by integrating their areas on the chromatograms, and arbitrarily expressing the obtained values as ratios with the peak ***c*** area, which was given the value 1 (Table 1). The data suggest that the relative areas of peaks were different in patients when compared to controls. Peak ***f*** was found markedly increased in glycan patterns of pHpt-P and sHpt-P (see also Figure 1, panels B and C versus panel A). A relational database was used to sort out the structures of Hpt major glycans, on the basis of their GU ±0.1. A number of possible structures could correspond to the values of GU we found. Thus, for example, ten different tri- and tetra-antennary glycans (with or without Fuc) are all reported to migrate as peak ***c***. These data confirm previous information on multiple structures of glycans of pHpt-P and pHpt-N, and indicate that also sHpt-P is glycosylated. In particular, the finding of higher amounts of peak ***f*** in the glycan pattern of sHpt-P, as compared with that of pHpt-P, suggests that sHpt-P diffuses from skin to blood, and comparative HPLC analysis of pHpt-P and pHpt-N might be a useful tool to monitor sHpt-P secretion into the skin.

**Table 1:** Relative amounts of the glycan peaks eluted by HPLC of pHpt-N, pHpt-P and sHpt-P.

Peak	GU^1^	pHpt-N^2^	pHpt-P^2^	sHpt-P^2^
***a***	7.97	0.31	0.36	0
***b***	8.06	0.39	0	0
***c***	9.88	1.00	1.00	1.00
***d***	10.68	0.25	0.34	0.46
***e***	11.25	0.40	0.40	0.23
***f***	11.80	0.40	0.71	1.78

1 GU value represents the peak elution as compared to that of 2-aminobenzamide-labelled oligomers of glucose. A mixture of standards (1 to 20 units of glucose) was used for the chromatography calibration.

2 The HPLC pattern of Hpt glycans, shown in Figure 1, was analyzed. The relative area of each peak is expressed as ratio with peak ***c*** area. Data from one experiment are shown. Inter-assay CV of each peak, from three separate experiments, was less than 5%.

### Analysis of Hpt glycans by lectins

**Figure 2:**
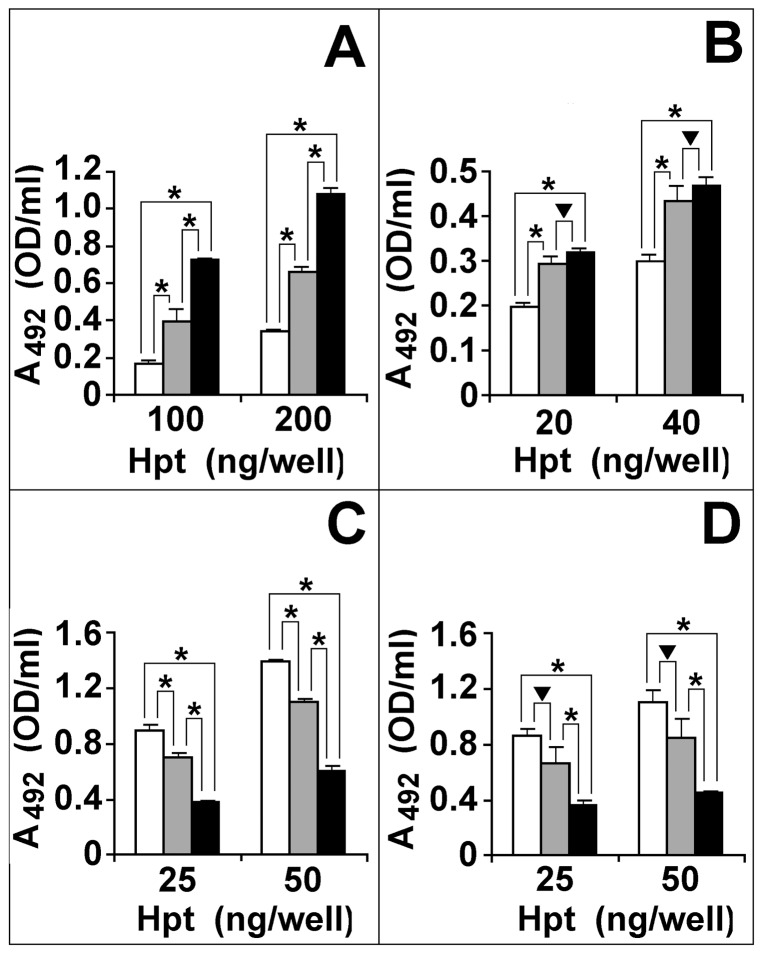
Binding of lectins to Hpt. The wells of a microtiter plate were coated with different amounts of pHpt-N (white bars), pHpt-P (grey bars), or sHpt-P (black bars). Solutions containing 1 µM biotinylated LTA (panel A), MAA (panel B), ConA (panel C), or SNA (panel D) were separately incubated into the wells. Avidin-HRP and hydrogen peroxide were used to develop color from OPD. Color intensity was determined by measuring the absorbance at 492 nm (A_492_). Five equal aliqouts of each sample were processed, and means ± SEM are shown. Asterisk: significant difference between the linked bars (P<0.0001). Triangle: not significant difference (P>0.05). Data from one experiment are shown. Inter-assay CV for each sample, from three separate experiments, was less than 5%.

ELISA with biotin-labeled lectins is a widely used technique for analysing the amounts of different structures of complex N-linked glycans. The lectins Concanavalin A (ConA, binding the mannose core of glycans), Sambucus nigra (SNA, binding S α2,6-linked to galactose, from here on named G), Maackia amurensis (MAA, binding S α2,3-linked to G) and Lotus tetragonolobus agglutinin (LTA, binding Fuc), were used to evaluate quantitative differences in their reactivity to the glycoforms of Hpt from plasma of patients and controls, and psoriatic skin scales. On the basis of our preliminary HPLC analysis, which suggested the presence in pHpt-P and sHpt-P of highly branched tetra-antennary glycans, pHpt-P and sHpt-P were expected to display lower reactivity to ConA whereas higher reactivity to LTA than pHpt-N. In fact, glycoforms with more highly branched glycans (as supposed in pHpt-P and sHpt-P) should have the mannose core more hindered by the glycan arms and, consequently, should react to ConA with decreased affinity. Furthermore, the HPLC data also suggested that more Fuc residues might be harboured on pHpt-P and sHpt-P glycoforms, which were therefore thought more reactive to LTA than pHpt-N glycoforms. Equal amounts of pHpt-N, pHpt-P, or sHpt-P were separately loaded into the wells of a microtiter plate, and processed for the binding of biotinylated lectins. As shown in Figure 2, the three distinct populations of Hpt glycoforms displayed significant differences in their affinity for each of the used lectins. In detail, sHpt-P and pHpt-P reacted worse than pHpt-N with biotin labelled ConA (25 ng: 42.78±0.4 and 78.1±3.4% respectively; 50 ng: 42.69±3.23 and 74.60±1.25%; Figure 2, Panel C). Moreover, sHpt-P and pHpt-P bound LTA more than pHpt-N (100 ng: 389.60±1.50 and 288.89±14.3% respectively; 200 ng: 316.90±7.60 and 187.13±7.26% respectively; Figure 2, Panel A), and MAA as well (20 ng: 159.40±1.84 and 146.91±7.23% respectively; 40 ng: 157.04±5.94 and 145.86±12.20% respectively; Figure 2, Panel B). Conversely, pHpt-P and sHpt-P reacted to SNA worse than pHpt-N (25 ng: 41.20±3.34 and 76.15±13.88% respectively; 50 ng: 41.52±0.78 and 76.64±12.77% respectively). These results confirm that pHpt-P and sHpt-P have more fucosylated and branched glycans than pHpt-N, and indicate that they display increased ratio of α2,3-linked with α2,6-linked S as compared with normal glycoforms.

### Mass spectrometry (MS) analysis of glycopeptides from trypsin treatment of Hpt

**Figure 3:**
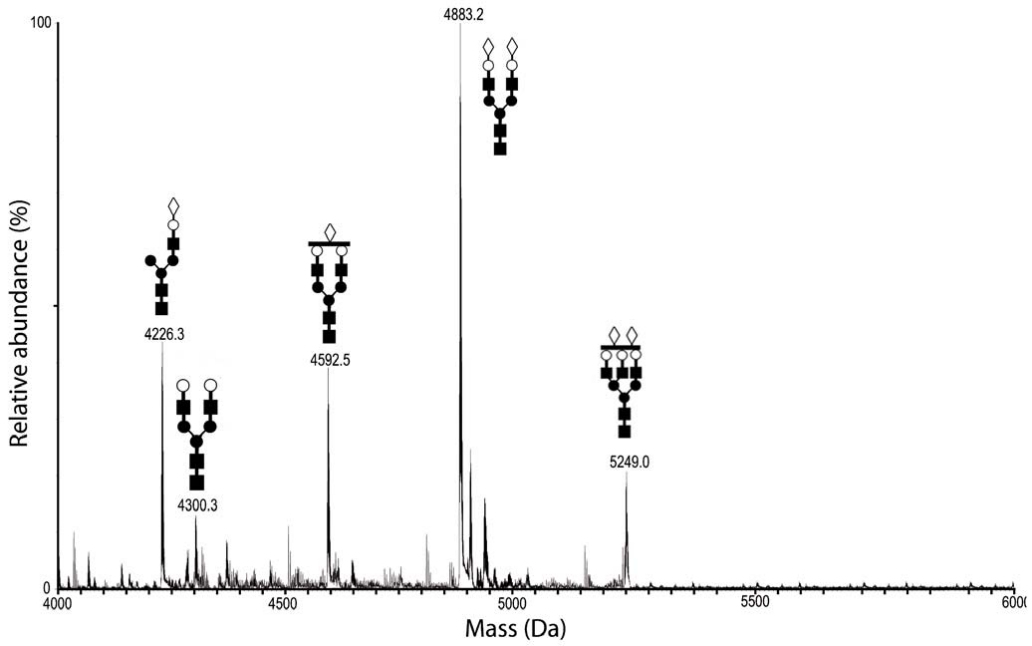
Mass spectrum of the P1 glycopeptide repertoire from skin of patients. Purified sHpt-P was digested by trypsin, and the resulting fragments were fractionated by UPLC and analyzed by ESI-MS. Positive ions of P1 (MVSHHNLTTGATLINEQWLLTTAK) glycopeptides from Hpt of skin of patients is shown. Glycopeptide peaks are indicated with their observed mass value (see Table 2) and chain structure. Peaks with mass attributable to non-glycosylated species were ignored.

**Figure 4:**
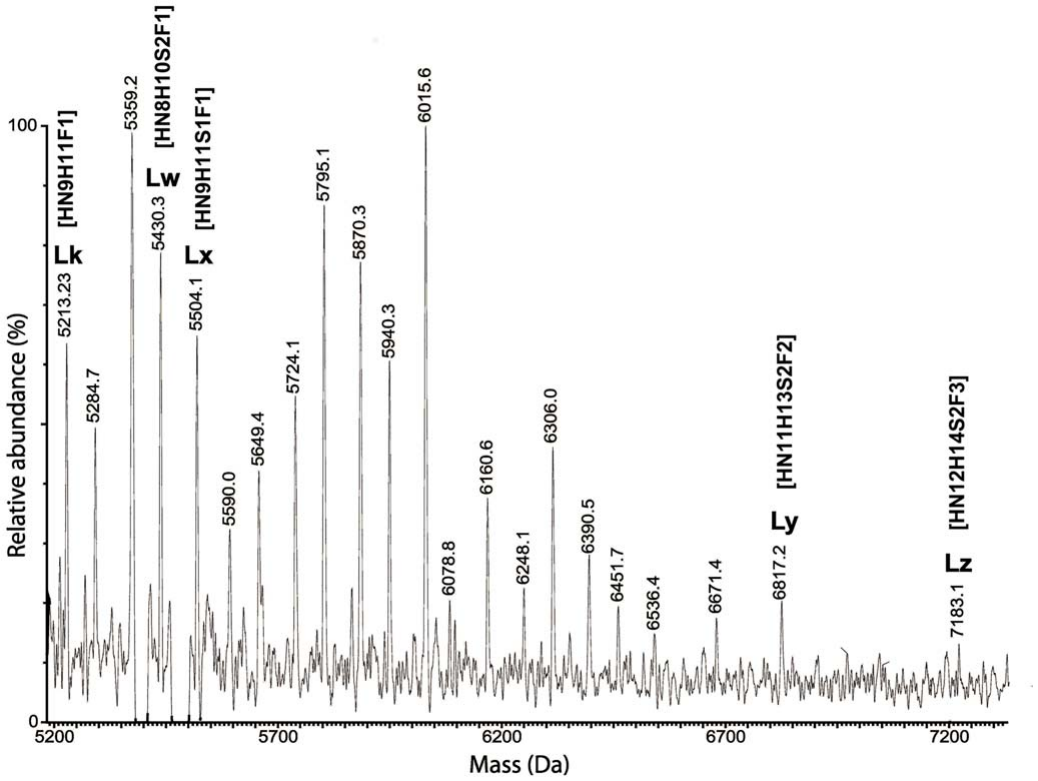
Mass spectrum of the P2 glycopeptide repertoire from skin of patients. Purified sHpt-P was digested by trypsin, and the resulting fragments were fractionated by UPLC and analyzed by ESI-MS. Positive ions of P2 (NLFLN HSENATAK) glycopeptides from Hpt of skin of patients is shown. Glycopeptide peaks are indicated with their observed mass value (see Table 3). The glycoforms found only in sHpt-P and not in pHpt-N and in pHpt-P are indicated. Peaks with mass attributable to non-glycosylated species were ignored.

**Figure 5:**
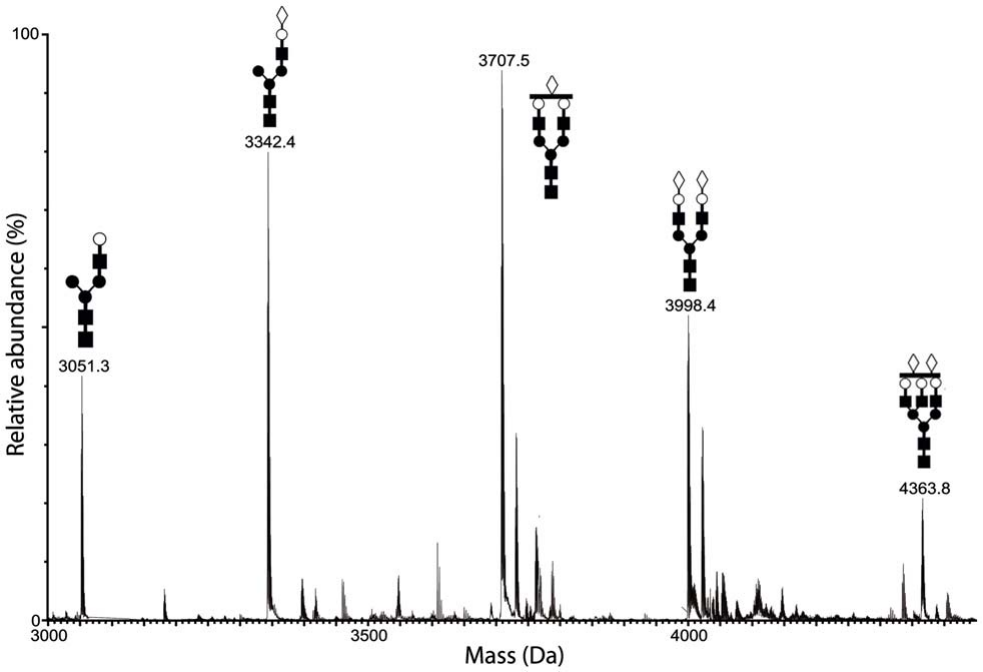
Mass spectrum of the P3 glycopeptide repertoire from skin of patients. Purified sHpt-P was digested by trypsin, and the resulting fragments were fractionated by UPLC and analyzed by ESI-MS. Positive ions of P3 (VVLHPNYSQVDIGLIK) glycopeptides from Hpt of skin of patients is shown. Glycopeptide peaks are indicated with their observed mass value (see Table 4) and chain structure. Peaks with mass attributable to non-glycosylated species were ignored.

MS analysis demonstrated that, in patients with moderate psoriasis, circulating Hpt displays both quantitative and qualitative changes in its glycan structures [16]. Hpt, purified from skin scales of psoriatic patients (sHpt-P) and cornified epidermal layer of healthy subjects (sHpt-N), was analyzed by MS following trypsin digestion, in order to further characterize glycan structures associated to Hpt from skin lesions, and to verify whether specific glycoforms in the skin of patients do exist. The digestion was predicted to yield 3 glycopeptides namely P1 (m/z 2679.39) carrying a single glycosylation site on N184, P2 (m/z 1458.74) with 2 glycosylation sites on N207 and N211, and P3 (m/z 1795.01) with a single glycosylation site on N241. The peptide mixtures were purified by gel filtration and processed for both liquid chromatography (LC)/MS and LC/MS/MS analysis. Many glycopeptides from both patients and controls were identified. MS spectra of the glycopeptides are reported in Figures 3–5, while Tables 2, 3, 4 report the observed masses, the corresponding predicted structures and the relative abundance of the established species. Peptides identification was based on the good agreement between experimental and theoretical mass values. Sialylated bi-antennary glycans were the major structures in Hpt from all sources. With regard to P1 (Figure 3), major glycans from sHpt-P were found to share structures with sHpt-N glycans (Table 2) or pHpt-P [16]. Most of the glycans found in the P2 repertoire (Figure 4; Table 3) from sHpt-P was also found in the previously reported glycan pattern of pHpt-P (18/29 signals found in pHpt-P) [16]. In particular, tetra-antennary N-glycans with one or two Fuc were observed in psoriatic Hpt (both skin and plasma), but not in control Hpt. Interestingly, the N-glycan repertoire of P2 from sHpt-P showed the presence of significant amounts of fucosylated structures and five glycan chains (harboured on species named as Lk, Lw, Lx, Ly and Lz) not detectable in the previously reported glycan pattern of pHpt-P [16]. These glycan structures were compatible with fucosylated but not fully sialylated glycan chain (Table 3). So, in agreement with the ELISA results shown above, the level of fucosylated glycans was found enhanced in sHpt-P (in particular in P2 glycoforms) compared to both pHpt-N and pHpt-P. Further, the Lw glycoform (not detectable in plasma) was also revealed in the P2 repertoire (Figure 4) from sHpt-N (Table 3), thus suggesting that these glycan structures are specifically associated with skin Hpt, and not with the pathology. Moreover, the overall level of fucosylated residues in sHpt-N was found lower than that from sHpt-P.

**Table 2:** Glycopeptides in the P1 repertoire of Hpt tryptic digest from skin of patients and controls, and their relative abundance.

Species	Observed Mass (Da)	Calculated Mass (Da)	Glycopeptide P1 (MVSHHNLTTGATLINEQWLLTTAK)^a^	Relative peak intensity^b^	
				Skin control	Skin scale Patients
M1	4227.02	4226.93	[NeuAc_1_Hex_1_HexNAc_1_+Man_3_GlcNAc_2_]	31.2±1.2	21.1±1.8
M2	4300.51	4300.97	[Hex_2_HexNAc_2_+Man_3_GlcNAc_2_]	4.8±0.3	2.9±0.9
M3	4591.56	4592.06	[NeuAc_1_Hex_2_HexNAc_2_+Man_3_GlcNAc_2_]	18.6±1.4	21.1±1.6
M4	4883.61	4883.16	[NeuAc_2_Hex_2_HexNAc_2_+Man_3_GlcNAc_2_]	46.4±1.8	47.8±3.1
M6	5248.66	5248.29	[NeuAc_2_Hex_3_HexNAc_3_+Man_3_GlcNAc_2_]	ND^c^	7.2±1.3

a The asparagine residue harbouring the glycan is underlined.

b Quantitative analysis for relative abundance of species was obtained taking into account all the observed multicharged ions. The values are expressed as means (from three technical replicates) ± standard deviations.

c ND = not detected. The ratio signal-to-noise = 3 was used as detection threshold.

Glycan species are indicated with the same nomenclature previously used for pHpt-P and pHpt-N [16].

**Table 3:** Glycopeptides in the P2 repertoire of Hpt tryptic digest from skin of patients and controls, and their relative abundance.

Species	Observed Mass (Da)	Calculated Mass (Da)	Glycopeptide P2 (NLFLNHSENATAK)^a^	Relative peak intensity^b^ ^c^	
				Skin Control	Skin scale Patients
L2	4993.82	4993.99	[NeuAc_1_Hex_2_HexNAc_2_+Man_3_GlcNAc_2_] + [Hex_2_HexNAc_2_+Man_3_GlcNAc_2_]	ND	ND
L3	5067.96	5068.03	[Hex_3_HexNAc_3_+Man_3_GlcNAc_2_] + [Hex_2_HexNAc_2_+Man_3_GlcNAc_2_]	7.6±1.0	ND
Lk	5213.23	5214.08	[Hex_3_HexNAc_3_+Man_3_GlcNAc_2_+Fuc_1_] + [Hex_2_HexNAc_2_+Man_3_GlcNAc_2_]	ND	4.9±0.8
L4	5284.93	5285.08	[NeuAc_1_Hex_1_HexNAc_1_+Man_3_GlcNAc_2_] + [NeuAc_1_Hex_2_HexNAc_2_+Man_3_GlcNAc_2_]	14.2±0.7	6.6±1.1
L5	5358.97	5359.12	[Hex_2_HexNAc_2_+Man_3_GlcNAc_2_] + [NeuAc_1_Hex_3_HexNAc_3_+Man_3_GlcNAc_2_]	13.4±1.3	5.6±1.1
Lw	5430.03	5431.14	[NeuAc_1_Hex_2_HexNAc_2_+Man_3_GlcNAc_2_+Fuc_1_] + [NeuAc_1_Hex_2_HexNAc_2_+Man_3_GlcNAc_2_]	5.4±0.9	13.3±1.2
Lx	5504.09	5505.18	[NeuAc_1_Hex_3_HexNAc_3_+Man_3_GlcNAc_2_+Fuc_1_] + [Hex_2_HexNAc_2_+Man_3_GlcNAc_2_]	ND	5.7±0.6
L6	5575.88	5576.18	[NeuAc_2_Hex_2_HexNAc_2_+Man_3_GlcNAc_2_] + [NeuAc_1_Hex_2_HexNAc_2_+Man_3_GlcNAc_2_]	19.7±1.5	ND
L7	5650.08	5650.21	[NeuAc_1_Hex_2_HexNAc_2_+Man_3_GlcNAc_2_] + [NeuAc_1_Hex_3_HexNAc_3_+Man_3_GlcNAc_2_]	6.8±0.3	5.6±1.2
L8	5722.18	5722.23	[NeuAc_2_Hex_2_HexNAc_2_+Man_3_GlcNAc_2_+Fuc_1_] + [NeuAc_1_Hex_2_HexNAc_2_+Man_3_GlcNAc_2_]	ND	3.5±0.4
L9	5796.11	5796.27	[NeuAc_1_Hex_3_HexNAc_3_+Man_3_GlcNAc_2_+Fuc_1_] + [NeuAc_1_Hex_2_HexNAc_2_+Man_3_GlcNAc_2_]	3.9±0.4	8.7±0.7
L10	5866.80	5867.27	[NeuAc_2_Hex_2_HexNAc_2_+Man_3_GlcNAc_2_] + [NeuAc_2_Hex_2_HexNAc_2_+Man_3_GlcNAc_2_]	9.9±0.9	4.9±0.8
L11	5941.74	5941.31	[NeuAc_2_Hex_3_HexNAc_3_+Man_3_GlcNAc_2_] + [NeuAc_1_Hex_2_HexNAc_2_+Man_3_GlcNAc_2_]	4.7±0.3	7.6±0.5
L12	6015.27	6015.35	[NeuAc_1_Hex_3_HexNAc_3_+Man_3_GlcNAc_2_] + [NeuAc_1_Hex_3_HexNAc_3_+Man_3_GlcNAc_2_]	6.5±0.2	9.1±0.8
L14	6161.34	6161.40	[NeuAc_1_Hex_3_HexNAc_3_+Man_3_GlcNAc_2_+Fuc_1_] + [NeuAc_1_Hex_3_HexNAc_3_+Man_3_GlcNAc_2_]	7.9±0.5	3.5±0.4
L16	6307.41	6307.46	[NeuAc_1_Hex_3_HexNAc_3_+Man_3_GlcNAc_2_+Fuc_1_] + [NeuAc_1_Hex_3_HexNAc_3_+Man_3_GlcNAc_2_+Fuc_1_]	ND	4.9±0.6
L18	6452.43	6452.49	[NeuAc_2_Hex_3_HexNAc_3_+Man_3_GlcNAc_2_+Fuc_1_] + [NeuAc_1_Hex_3_HexNAc_3_+Man_3_GlcNAc_2_]	ND	2.1±0.8
L21	6672.36	6672.60	[NeuAc_1_Hex_4_HexNAc_4_+Man_3_GlcNAc_2_+Fuc_1_] + [NeuAc_1_Hex_3_HexNAc_3_+Man_3_GlcNAc_2_+Fuc_1_]	ND	6.6±0.1
Ly	6817.23	6818.65	[NeuAc_1_Hex_4_HexNAc_4_+Man_3_GlcNAc_2_+Fuc_2_] + [NeuAc_1_Hex_3_HexNAc_3_+Man_3_GlcNAc_2_+Fuc_1_]	ND	6.0±0.6
Lz	7183.13	7183.79	[NeuAc_1_Hex_4_HexNAc_4_+Man_3_GlcNAc_2_+Fuc_2_] + [NeuAc_1_Hex_4_HexNAc_4_+Man_3_GlcNAc_2_+Fuc_1_]	ND	0.7±0.2
L29	7329.44	7329.85	[NeuAc_1_Hex_4_HexNAc_4_+Man_3_GlcNAc_2_+Fuc_2_] + [NeuAc_1_Hex_4_HexNAc_4_+Man_3_GlcNAc_2_+Fuc_2_]	ND	0.4±0.2

a The asparagine residue harbouring the glycan is underlined.

b Quantitative analysis for relative abundance of species was obtained taking into account all the observed multicharged ions. The values are expressed as means (from three technical replicates) ± standard deviations.

c ND = not detected. The ratio signal-to-noise = 3 was used as detection threshold.

Glycan species are indicated with the same nomenclature previously used for pHpt-P and pHpt-N [16].

**Table 4:** Glycopeptides in the P3 repertoire of Hpt tryptic digest from skin of patients and controls, and their relative abundance.

Species	Observed Mass (Da)	Calculated Mass (Da)	Glycopeptide P3 (VVLHPNYSQVDIGLIK)^a^	Relative peak intensity^b^	
				Skin Control	Skin scale Patients
N1	3051.15	3051.45	[Hex_1_HexNAc_1_+Man_3_GlcNAc_2_]	10.8±0.6	15.4±1.6
N2	3342.20	3342.55	[NeuAc_1_Hex_1_HexNAc_1_+Man_3_GlcNAc_2_]	19.9±1.8	26.9±2.8
N4	3707.26	3707.68	[NeuAc_1_Hex_2_HexNAc_2_+Man_3_GlcNAc_2_]	26.3±1.1	29.9±2.2
N7	3998.28	3998.78	[NeuAc_2_Hex_2_HexNAc_2_+Man_3_GlcNAc_2_]	33.6±1.9	19.2±2.0
N10	4363.30	4363.91	[NeuAc_2_Hex_3_HexNAc_3_+Man_3_GlcNAc_2_]	9.4±1.0	8.5±1.3

a The asparagine residue harbouring the glycan is underlined.

b Quantitative analysis for relative abundance of species was obtained taking into account all the observed multicharged ions. The values are expressed as means (from three technical replicates) ± standard deviations.

Glycan species are indicated with the same nomenclature previously used for pHpt-P and pHpt-N [16].

In the P3 repertoire (Figure 5), the glycoform population of sHpt-P (5 species) was less heterogeneous than that previously found in pHpt-P (14 species) [16]. Minor differences in relative abundance for some glycans or in glycan structures, between sHpt-P and sHpt-N, are shown in Table 4.

The glycan structures were also analyzed in Hpt purified from a pool of plasma of patients (pHpt-R) that, after treatment with the anti-TNF- α drug Adalimumab for 16 weeks, displayed disease remission (PASI = 3.9±1.2). No significant differences were found in the P1 repertoire from pHpt-R (Table 5), in comparison with that previously reported in pHpt-P [16], while fucosylated and highly branched glycans were found in significantly lower amounts in the P2 and P3 repertoire from pHpt-R (Table 6 and 7, respectively). As TNF-α was shown to induce the expression of α2,6-sialyl transferase and fucosyl transferase [28], [29], our results suggest a potential inhibitory effect of Adalimumab on these enzyme activities.

**Table 5:** Glycopeptides in the P1 repertoire of Hpt tryptic digest from plasma of patients in disease remission.

Species	Observed Mass (Da)	Calculated Mass (Da)	Glycopeptide P1 (MVSHHNLTTGATLINEQWLLTTAK)^a^	Relative peak intensity^b^
M1	4227.02	4226.93	[NeuAc_1_Hex_1_HexNAc_1_+Man_3_GlcNAc_2_]	25.8±1.5
M2	4300.51	4300.97	[Hex_2_HexNAc_2_+Man_3_GlcNAc_2_]	5.4±0.8
M3	4591.56	4592.06	[NeuAc_1_Hex_2_HexNAc_2_+Man_3_GlcNAc_2_]	23.3±1.3
M4	4883.61	4883.16	[NeuAc_2_Hex_2_HexNAc_2_+Man_3_GlcNAc_2_]	41.3±2.6
M6	5248.66	5248.29	[NeuAc_2_Hex_3_HexNAc_3_+Man_3_GlcNAc_2_]	5.2±0.8

a The asparagine residue harbouring the glycan is underlined.

b Quantitative analysis for relative abundance of species was obtained taking into account all the observed multicharged ions. The values are expressed as means (from three technical replicates) ± standard deviations.

Glycan species are indicated with the same nomenclature previously used for pHpt-P and pHpt-N [16].

**Table 6:** Glycopeptides in the P2 repertoire of Hpt tryptic digest from plasma of patients in disease remission.

Species	Observed Mass (Da)	Calculated Mass (Da)	Glycopeptide P2 (NLFLNHSENATAK)^a^	Relative peak intensity^b^
L2	4993.82	4993.99	[NeuAc_1_Hex_2_HexNAc_2_+Man_3_GlcNAc_2_] + [Hex_2_HexNAc_2_+Man_3_GlcNAc_2_]	3.5±0.7
L3	5067.96	5068.03	[Hex_3_HexNAc_3_+Man_3_GlcNAc_2_] + [Hex_2_HexNAc_2_+Man_3_GlcNAc_2_]	3.9±0.5
L4	5284.93	5285.08	[NeuAc_1_Hex_1_HexNAc_1_+Man_3_GlcNAc_2_] + [NeuAc_1_Hex_2_HexNAc_2_+Man_3_GlcNAc_2_]	8.4±1.0
L5	5358.97	5359.12	[Hex_2_HexNAc_2_+Man_3_GlcNAc_2_] + [NeuAc_1_Hex_3_HexNAc_3_+Man_3_GlcNAc_2_]	8.8±0.7
Lw	5430.03	5431.14	[NeuAc_1_Hex_2_HexNAc_2_+Man_3_GlcNAc_2_+Fuc_1_] + [NeuAc_1_Hex_2_HexNAc_2_+Man_3_GlcNAc_2_]	12.2±0.8
Lx	5504.09	5505.18	[NeuAc_1_Hex_3_HexNAc_3_+Man_3_GlcNAc_2_+Fuc_1_] + [Hex_2_HexNAc_2_+Man_3_GlcNAc_2_]	6.5±0.6
L6	5575.88	5576.18	[NeuAc_2_Hex_2_HexNAc_2_+Man_3_GlcNAc_2_] + [NeuAc_1_Hex_2_HexNAc_2_+Man_3_GlcNAc_2_]	7.6±0.2
L7	5650.08	5650.21	[NeuAc_1_Hex_2_HexNAc_2_+Man_3_GlcNAc_2_] + [NeuAc_1_Hex_3_HexNAc_3_+Man_3_GlcNAc_2_]	4.2±0.3
L9	5796.11	5796.27	[NeuAc_1_Hex_3_HexNAc_3_+Man_3_GlcNAc_2_+Fuc_1_] + [NeuAc_1_Hex_2_HexNAc_2_+Man_3_GlcNAc_2_]	6.1±0.5
L10	5866.80	5867.27	[NeuAc_2_Hex_2_HexNAc_2_+Man_3_GlcNAc_2_] + [NeuAc_2_Hex_2_HexNAc_2_+Man_3_GlcNAc_2_]	9.4±0.5
L11	5941.74	5941.31	[NeuAc_2_Hex_3_HexNAc_3_+Man_3_GlcNAc_2_] + [NeuAc_1_Hex_2_HexNAc_2_+Man_3_GlcNAc_2_]	10.2±1.0
L14	6161.34	6161.40	[NeuAc_1_Hex_3_HexNAc_3_+Man_3_GlcNAc_2_+Fuc_1_] + [NeuAc_1_Hex_3_HexNAc_3_+Man_3_GlcNAc_2_]	9.3±1.5
L16	6307.41	6307.46	[NeuAc_1_Hex_3_HexNAc_3_+Man_3_GlcNAc_2_+Fuc_1_] + [NeuAc_1_Hex_3_HexNAc_3_+Man_3_GlcNAc_2_+Fuc_1_]	4.8±0.5
L21	6672.36	6672.60	[NeuAc_1_Hex_4_HexNAc_4_+Man_3_GlcNAc_2_+Fuc_1_] + [NeuAc_1_Hex_3_HexNAc_3_+Man_3_GlcNAc_2_+Fuc_1_]	5.1±0.8

a The asparagine residue harbouring the glycan is underlined.

b Quantitative analysis for relative abundance of species was obtained taking into account all the observed multicharged ions. The values are expressed as means (from three technical replicates) ± standard deviations. Glycan species are indicated with the same nomenclature previously used for pHpt-P and pHpt-N [16].

**Table 7:** Glycopeptides in the P3 repertoire of Hpt tryptic digest from patients in disease remission.

Species	Observed Mass (Da)	Calculated Mass (Da)	Glycopeptide P3 (VVLHPNYSQVDIGLIK)^a^	Relative peak intensity^b^
N1	3051.15	3051.45	[Hex_1_HexNAc_1_+Man_3_GlcNAc_2_]	14.7±1.6
N2	3342.20	3342.55	[NeuAc_1_Hex_1_HexNAc_1_+Man_3_GlcNAc_2_]	27.0±2.3
N4	3707.26	3707.68	[NeuAc_1_Hex_2_HexNAc_2_+Man_3_GlcNAc_2_]	28.9±1.6
N7	3998.28	3998.78	[NeuAc_2_Hex_2_HexNAc_2_+Man_3_GlcNAc_2_]	22.1±1.6
N10	4363.30	4363.91	[NeuAc_2_Hex_3_HexNAc_3_+Man_3_GlcNAc_2_]	7.3±0.9

a The asparagine residue harbouring the glycan is underlined.

b Quantitative analysis for relative abundance of species was obtained taking into account all the observed multicharged ions. The values are expressed as means (from three technical replicates) ± standard deviations.

Glycan species are indicated with the same nomenclature previously used for pHpt-P and pHpt-N [16].

## Discussion

Identification of clinically important protein biomarkers with possible glycosylation alteration is an expanding area of research that can improve diagnosis of psoriasis. Our previous results showed that Hpt from psoriatic patients contains quantitative and qualitative glycoform variants from those circulating in normal conditions [16]. In this study, we provide the first evidence of differences between circulating and skin Hpt. Here we report that the HPLC patterns of glycans from pHpt-P and sHpt-P were different from that of glycans from pHpt-N. In particular, some pHpt-P glycans, as compared with pHpt-N glycans displaying the same GU value, were present at lower level, or missing whereas other glycans were more abundant. The observed quantitative differences were enhanced in the pattern from skin Hpt. In this pattern we detected greater amounts of two major peaks, and material containing highly branched glycans. These data provide evidence on changes in glycan structures linked to Hpt of patients with psoriasis, and suggest that disease condition enhances glycan branching and Fuc content. In this frame, the finding that quantitatively rather than qualitatively changes increase in glycans of skin Hpt, as compared with those of pHpt-P, led us to argue that glycoforms of the former protein population might diffuse from epidermis to blood thus modifying the glycoform composition of circulating Hpt. Hpt with altered glycosylation pattern might be produced by keratinocytes [13] in the skin at the site of inflammation and secreted into the plasma. Our hypothesis on changes and source of psoriasis-associated Hpt glycoforms was strengthened by results from experiments with lectins. In fact, besides the HPLC experiments, also the assays of Hpt reactivity to specific lectins indicate that pHpt-P contains more highly branched glycans and higher Fuc level than pHpt-N, and such a difference increases with skin Hpt. The MS analysis of skin Hpt confirmed that clear differences in the N-glycan repertoire do exist between psoriatic and healthy subjects. Actually P2 glycoforms with fucosylated tri- or tetra-antennary N-glycans (Lk, Lw, Lx, Ly, and Lz), that were not previously detected in pHpt-P and pHpt-N [16], were detected in sHpt-P. The finding of the glycoform Lw also in the glycan repertoire of sHpt-N suggests that its glycan structure is specifically associated with skin Hpt, and not with the disease. Moreover, the amount of fucosylated residues was higher in sHpt-P than in sHpt-N. Significant increases in Fuc levels and glycan branching in Hpt have been found to be associated with ovarian cancer [21], [30], lung cancer [31], pancreatic cancer [23], colon cancer [32] and hepatocellular carcinoma [33]. A body of information is available about highly branched glycans and increased Fuc level in other acute phase glycoproteins in several diseases. Therefore, it is conceivable that such glycans might play a role during inflammation. On the other side, changes in normal glycan structures might be associated with loss of normal function. These two consequences of glycan modification might not be alternative, and both might occur in disease. As a matter of fact, glycans of Hpt are known to be engaged in binding haemoglobin [34], but pHpt-P was found displaying lower binding activity than pHpt-N [15].

Our results also show that Hpt from patients contains lower amount in sialic acid residues α2,6-linked to galactose than normal Hpt. This type of glycosidic bond is required for immunosuppression of lymphocytes B [35]. Conversely, in psoriasis, Hpt contains higher amount in sialic acid residues α2,3-linked to galactose than normal Hpt. This change in the type of glycosidic bond likely occurs on tri-antennary chains because α2,3-sialyltrasferase shows a much higher affinity than α2,6-sialyltransferase for arms growing on branched glycans [36]. Therefore increase in the amount of α2,3-linked sialic acid well agrees with increase in branching of Hpt from patients. These features are both known to promote the binding of glycoproteins to Galectin-3 [37], [38]. Hpt glycoforms which binds Galectin-3 were actually detected [39], [40]. Galectins is known to play regulatory roles in inflammation, immunity and cancer [41]–[44]. In particular, Galectin-3 plays a number of roles on lymphocytes T such as modulation of cell adhesion and induction of apoptosis [45], and secretion of IL-6 [46]. Galectin-3 is abundantly expressed also by keratinocytes and other epithelial cell, and was suggested to inhibit ERK and stimulate Akt [47]. It is therefore conceivable that Hpt, in the skin of patients with psoriasis, might participate to mechanisms regulating the immune response. Also Galectin-1 was found bound to Hpt glycoforms, that were present at increased levels in sera of patients with breast cancer [48]. These glycoforms, containing N-linked glycans with less terminal sialic acids and more arms than normal glycoforms, had different trafficking, as compared to non-bound glycoforms, after their endocytosis into macrophages [48]. Further analyses are needed to evaluate whether skin Hpt interacts with Galectin-1 or Galectin-3 in the skin, or targets cells involved in the autoimmune response in psoriasis, and to disclose molecular mechanisms in which the specific glycoforms we detected might participate (o fail to participate).

The analysis of glycan structures of pHpt-R showed the presence of a lower amount of fucosylated and highly branched structures, when compared with the previously reported N-glycan repertoire of pHpt-P [16]. Furthermore, the N-glycan repertoire of pHpt-R revealed the presence of two glycoforms (namely Lw and Lx). Since these glycan structures were present in sHpt-P and sHpt-N, but not in pHpt-N and pHpt-P [16], this result suggests that the anti-TNF-α treatment might support Lw and Lx production in the anti-inflammatory response.

In conclusion, three glycoforms (Lk, Ly and Lx) were specifically detected only in psoriatic skin, and might be used as markers of the pathology, although it remains to clarify whether these glycoforms are specific for psoriasis or are also associated with skin diseases different from psoriasis. However, a more detailed analysis of changes in the glycan structures of Hpt from both plasma and skin is required to assess whether these changes are associated with disease progression and/or relapse.

## Materials and Methods

### Materials

Chemicals of the highest purity, bovine serum albumin (BSA), human Hpt (mixed phenotypes: Hpt 1-1, Hpt 1–2, Hpt 2–2), rabbit anti-human Hpt IgG, goat anti-rabbit horseradish peroxidase-conjugated (GAR-HRP) IgG, *o*-phenylenediamine (OPD), 2-aminobenzamide, N-glycosidase from *Flavobacterium meningosepticum* (PNGase F, E.C. 3.5.1.52), phenylmethanesulfonyl fluoride (PMSF), and Avidin conjugated with horseradish peroxidase (Avidin-HRP), were purchased from Sigma-Aldrich (St. Louis, MO, USA). Organic solvents were purchased from Romil (Cambridge, UK), and polystyrene 96-wells microtiter plates from Nunc (Roskilde, Denmark). 2-aminobenzamide-labelled glycans, and glucose oligomers for HPLC calibration were obtained from Ludger (Culham Science Center, Oxfordshire, UK). Discovery DPA-6S solid-phase extraction columns were purchased from Supelco (Bellefonte, PA, USA), and the TSK-gel Amide-80 column was from Tosoh Bioscience (Stuttgart, Germany). Sephacryl S-200, HiTrap Blue HP polypropylene column, and CNBr-activated Sepharose CL-4B of GE Healthcare (Uppsala, Sweden) were used. The Biotin-labelled lectins LTA, MAA, SNA, and ConA were purchased from EY laboratories (San Mateo, CA, USA).

### Ethic statements

The present investigation conforms to the principles outlined in the declaration of Helsinki, and it was approved by the Ethics Committee of the University of Naples Federico II. Patients and healthy subjects were enrolled in the study after their informed consent.

### Patients

Patients (n = 5) with psoriasis vulgaris and control subjects (n = 9), matched for age (range: 30-40 years) and cardiovascular risk factors were enrolled after their written informed consent. In particular patients with PASI>15 (disease of moderate entity), not taking anti-inflammatory drugs, were included in the study. Patients in disease remission (average PASI =  3.9±1.2; n = 7), after anti-TNF-α treatment with adalimumab for 16 weeks, were also enrolled. The treatment produced a decrease of PASI of 82.0±4.3%. No subject had any symptom or laboratory finding of kidney, liver or thyroid dysfunction, infection, diabetes, cardiovascular disease, or malignancy.

### Hpt purification

Hpt was purified from pooled plasma (each sample contributing in the pool with equal volume) of patients with moderate psoriasis (n = 5), patients under remission (n = 7), or controls (n = 9) as previously reported [16]. Hpt was also isolated from pooled skin scales (2.2 g, each sample contributing in the pool with equal weight) of psoriatic patients (n = 6) or from cornified epidermal layer (4.4 g) of healthy subjects (n = 14) as follows. The scales were cut into small pieces, and ground in liquid nitrogen by a mortar. The powder was recovered in 10 volumes of ice-cold solution containing 0.5% (w/v) deoxycholate, 250 mM NaCl, 2 mM EDTA, and 1 mM PMSF in 50 mM Tris-HCl at pH 8.0. The resulting homogenate was centrifuged (30 min; 20,000 g; 0°C), and the supernatant was applied onto anti-Hpt IgG affinity column chromatography (1.5×5 cm) according to manufacturer's instructions. After sample loading, the column was extensively washed by 50 mM sodium phosphate at pH 7.4 to remove unbound material. Column-bound Hpt was eluted with 20 ml of 0.1 M glycine-HCl at pH 3.0 neutralized with 10 µl of 1 M Tris, and then subjected to SDS-PAGE analysis for purity check, as previously reported [7], before further experiments.

### PNGase F digestion of Hpt and preparation of fluorescent glycans

PNGase F digestion was carried out on Hpt purified from plasma and skin of patients or plasma of controls (pHpt-P, sHpt-P, and pHpt-N respectively). Hpt samples (50 µg), in 45 µl of 10 mM sodium phosphate at pH 7.4, were mixed with 2 µl of 5% SDS and 3 µl of 1.6 M β-mercaptoethanol, and then heated at 100°C for 5 min. The solution was supplemented with 5 µl of 15% Triton X-100 and 10 µl of 500 units/ml PNGase F. Following incubation overnight at 37°C, the samples were heated at 100°C for 5 min, and then analyzed by SDS-PAGE to control effective deglycosylation. SDS-PAGE analysis of glycosylated and deglycosylated Hpt showed that the protein purification procedure and the β subunit deglycosylation were effective, and provided evidence that only such a subunit harbours.

N-linked glycans. The free oligosaccharides were labelled by conjugating the fluorescent probe 2-aminobenzamide to their reducing end through reductive amination reaction. The reagent mixture for fluorescent labelling was prepared by dissolving 2-aminobenzamide and sodium cyanoborohydride (0.24 and 0.68 M final concentration, respectively) in a solution containing 3.5 ml of DMSO and 2 ml of glacial acetic acid. An aliquot of 5 µl from this mixture was added to the solution containing glycans with deglycosylated Hpt. After 3 h at 70°C, the labelled oligosaccharides were purified using a Discovery DPA-6S column for solid-phase extraction. This column, equilibrated with 2 ml of CH_3_CN, was loaded with the sample under gravity at room temperature, and washed with 4 ml of 99% CH_3_CN, and then 0.5 ml of 97% CH_3_CN. The labelled glycans were eluted with 1 ml of 0.5 M formic acid. The glycan solution was dried under vacuum, and re-dissolved by 40 µl of 87.5 mM ammonium formate in CH_3_CN. Aliquots of 20 µl were used for HPLC analysis.

### HPLC

Purified labelled glycans were fractionated by normal phase HPLC using a TSK-gel Amide-80 column (4.6×250 mm) essentially according to He et al., 2006 [27]. The fluorescence detector was set at λ_EX_ = 360 nm and λ_EM_ = 425 nm. The chromatography was performed at 30°C. CH_3_CN and 250 mM ammonium formate at pH 4.4 were used for the mobile phase. Elution was carried out at 0.4 ml/min, by simultaneous linear gradients of CH_3_CN (65 to 35%) and formate (35 to 65%) in 75 min. A mixture of labelled glucose and glucose oligomers (1 to 20 glucose units, namely GU) was used for calibration. The GU ladder was used as a scale for elution of the glycans from Hpt. Standard labelled glycans (NeuAc_2_Hex_2_HexNAc_2_+Man_3_GlcNAc_2_; NeuAc_1_Hex_2_HexNAc_2_+Man_3_GlcNAc_2_; NeuAc_2_Hex_2_HexNAc_2_+Man_3_GlcNAc_2_+Fuc_1;_ NeuAc_3_Hex_3_HexNAc_3_+Man_3_GlcNAc_2_) served as chromatography controls. The database used in this work was GlycoBase (http://glycobase.ucd.ie).

### Glycan assay by Lectins

Purified Hpt was analyzed for its reactivity to different lectins, by ELISA. Four lectins (LTA, MAA, SNA and ConA) were used to detect glycan specific structures. LTA actually binds Fuc α1,6- or α1,3-linked to N-acetylglucosamine in arm or dichitobiose respectively. MAA can detect glycans containing S α2,3-linked to G, whereas SNA binds S α2,6-linked to G. ConA is commonly used to detect the mannose core of N-linked complex glycans, and such a detection is recognized to be negatively correlated with the number of arms (i.e. substitutions of mannose residues) because more arms more steric hindrance shields the mannose core. Microtiter wells were incubated with different amount of Hpt in coating buffer (7 mM Na_2_CO_3_, 17 mM NaHCO_3_, 1.5 mM NaN_3_, pH 9.6) for 2 h at room temperature. In detail, 100 and 200 ng/well of Hpt were used for LTA binding, 20 and 40 ng/well for MAA, and 25 and 50 ng/well for ConA or SNA. Wells not coated with Hpt were used as control. Unbound Hpt was removed by three washes with 130 mM NaCl and 0.05% (w/v) Tween-20 in 20 mM Tris-HCl at pH 7.3, and further three washes with 500 mM NaCl in 20 mM Tris-HCl at pH 7.3. The possible sites of protein absorption were then blocked by incubation (1 h, 37°C) with 130 mM NaCl, 20 mM Tris-HCl, pH 7.4 (TBS) containing 2% (w/v) Tween 20. After extensive washing, as above, the wells were loaded with either biotin-labelled lectin (1 µM in binding buffer, that was TBS containing 1 mM CaCl_2_, 1 mM MgCl_2_, and 0.05% Tween-20), and incubated overnight at 4°C. After extensive washing, the wells were loaded with Avidin-HRP (1∶12,000 dilution in binding buffer) and incubated for 1 h at 37°C. The lectin binding was detected by measuring at 492 nm the colour developed from OPD in the presence of hydrogen peroxide. Wells, coated with the same aliquots of Hpt used for lectin binding, were incubated with rabbit anti-Hpt IgG and GAR-HRP according to a published procedure [49], as controls of protein amounts used for assaying comparative reactivity of pHpt-N, pHpt-P, and sHpt-P for any lectin used.

### Mass spectrometry

MS analysis of Hpt, purified from skin scales of patients, or from cornified epidermal layer of healthy donors, or plasma of patients in remission, was carried out. In particular, purified Hpt was analyzed by MS, after trypsin digestion and fragments fractionation by UPLC, as previously reported [16].

### Decoding of sugar composition into structure of complex N-linked glycans

Three N-linked glycopeptides were expected to result from Hpt (accession number: P00738) digestion by trypsin, namely glycosylated fragments P1, P2, and P3 (containing the amino acid sequences 179–202, 203–215, and 236–251, respectively). The data on the carbohydrate composition were obtained as follows. The mass of a given glycopeptide was considered to be the sum of glycan mass plus peptide mass. Therefore, the masses of P1 (2679.39), P2 (1458.74), and P3 (1795.01) were subtracted from each glycopeptides mass, and the three resulting values were analyzed separately for their content in units of hexose (Hex, H for acronym), N-acetylhexosamine (HexNAc, HN for acronym), deoxyhexose (dHex), and N-acetylneuraminic acid (NeuAc, or S for acronym as already mentioned).

Composition decoding was done by a step-to-step building up procedure starting from the reducing end of the glycan, according to current information and nomenclature for structures of N-linked complex glycans. Thus, for example, the composition unit HexNAc2Hex3 was considered to account for dichitobiose (two residues of N-acetylglucosamine joined by a β1–4 glycosidic bond), followed by a mannose core (β1–4 linked mannose, which is 3-O- and 6-O-substituted with two α mannose), and named Man3GlcNAc2. Further steps were substitutions of the mannose core with HexNAc, which is named antenna, or bisecting if linked to terminal or dichitobiose-linked mannose, respectively. Each arm was elongated with Hex (galactose). NeuAc, when present, was considered to be at the terminal end of the arm. Substitution of dichitobiose or arm(s) with Fuc accounted for dHex units. Where Fuc is linked (on dichitobiose or antenna) and whether NeuAc is α2,3 or α2,6 linked to Hex was not assessed in this study. Thus, a mass compatible with, for example, Hex5HexNAc4NeuAc1 was expressed as (NeuAc1Hex2HexAc2+Man3GlcNAc2) in MS analysis or HN4H5S1 for brevity. Similarly, the carbohydrate composition from two glycans of P2, e.g. (NeuAc1Hex1HexNAc1+Man3GlcNAc2) together with (NeuAc1Hex2HexNAc2+Man3GlcNAc2) was also expressed as HN7H9S2.

### Statistics

In the HPLC experiments, at least three separate preparations of Hpt glycans were used, and inter-assay CV for homologous peaks of pHpt-N, pHpt-P, or sHpt-P was calculated. In assays of lectin binding, experiments with three separate preparations of pHpt-N, pHpt-P, and sHpt-P were carried out, and five equal aliquots of each preparation were analyzed. In these assays, data from each experiment were expressed as means ± SEM. Statistical differences were determined using t-test or, where appropriate, one-way ANOVA, followed by Tukey's test for multiple comparisons (GraphPad Software Inc., San Diego, CA). Differences were considered statistically significant when the two-sided *P* value was less than 0.05.
